# Safe discontinuation of nilotinib in a patient with chronic myeloid leukemia: a case report

**DOI:** 10.1186/1752-1947-8-295

**Published:** 2014-09-06

**Authors:** Giovanni Caocci, Marianna Greco, Giorgio La Nasa

**Affiliations:** 1 Bone Marrow Transplant Center, "R Binaghi" Hospital, Via Is Guadazzonis 3, 09126 Cagliari, Italy; 2 Hematology Unit, Department of Medical Sciences, University of Cagliari, Via Is Guadazzonis 3, 09126 Cagliari, Italy

**Keywords:** Chronic myeloid leukemia, Imatinib, Killer immunoglobulin-like receptors, Nilotinib, Tyrosine kinase inhibitor discontinuation

## Abstract

**Introduction:**

Although there is a considerable amount of data in the literature on safe discontinuation of first-generation tyrosine kinase inhibitor therapy in patients with chronic myeloid leukemia, little is known about discontinuation of second-generation tyrosine kinase inhibitor therapy. Most previous studies have been focused on dasatinib, and the few cases of nilotinib withdrawal that have been reported had a median follow-up of 12 months. To the best of our knowledge, the present report is the first to describe nilotinib withdrawal with 30 months of follow-up.

**Case presentation:**

We report the case of a 64-year-old Caucasian man diagnosed with chronic-phase chronic myeloid leukemia in April 2005. After 4 years of treatment with imatinib, he became intolerant to the drug and was switched to nilotinib. Two years later, he decided to stop nilotinib. Undetectable molecular response persisted for 30 months after discontinuation of the drug.

**Conclusion:**

Our present case suggests that nilotinib withdrawal is safe for patients with chronic myeloid leukemia who achieve a stable undetectable molecular response. Our patient was homozygous for killer immunoglobulin-like receptor haplotype A, previously reported to be a promising immunogenetic marker for undetectable molecular response. We recommend additional studies to investigate patient immunogenetic profiles and their potential role in complete response to therapy.

## Introduction

One of the most highly debated challenges physicians face when treating patients with chronic myeloid leukemia (CML) is to decide whether tyrosine kinase inhibitor (TKI) therapy can be stopped or must be continued indefinitely. The recent European LeukemiaNet (ELN) guidelines for the management of CML recommend that patients who respond optimally to TKI treatment be continued on it indefinitely at the standard suggested dose [1].

Researchers in several studies have reported safe discontinuation of first-generation TKI imatinib. Overall, these studies show a probability of stable undetectable molecular response (MR) of approximately 40% at 24 months. Factors associated with sustained response are lower Sokal risk score, duration of imatinib treatment, previous interferon therapy, early major MR and duration of undetectable MR [2-4].

However, little is known to date about discontinuation of second-generation TKI therapy. In one study of 34 patients with CML, the investigators reported the feasibility of discontinuing both nilotinib and dasatinib with a median follow-up of 12 months [5]. In a recent preliminary report, researchers described safe withdrawal of dasatinib with a median follow-up of 6 months in 27 patients previously treated with imatinib [6]. Additionally, in three case reports of a total of six patients with median follow-up ranging from 12 to 27 months, the authors showed safe discontinuation in three of the five patients treated with dasatinib and in one patient treated with nilotinib [7-9]. Our report concerns an imatinib-intolerant patient who achieved undetectable MR with nilotinib. The patient was in undetectable MR4.5 at 30 months after stopping the drug and remains so at the time of this report.

## Case presentation

Our patient was a 64-year-old Caucasian man diagnosed with chronic phase CML in April 2005. Peripheral blood tests showed hemoglobin of 13.5g/dl, white blood cell count of 17.6×10^9^/L and platelet count of 402×10^9^/L. No organomegaly was observed. Cytogenetic analysis of the bone marrow aspirate revealed a 46,XY,t(9;22)(q34;q11.2) karyotype in 100% of metaphases. By qualitative PCR, we detected the sole presence of the b3a2 transcript. According to the patient’s Sokal and Hasford scores, he was classified as being in an intermediate risk class. He was started on imatinib 400mg/day. After 6 months, he had achieved a complete cytogenetic response and MR3 (BCR-ABL1≤0.01%). Four years later, the patient became intolerant to imatinib (grade 3 diarrhea, probably due to the irritant effect of this drug on intestinal mucosa) and was switched to nilotinib. Although the recommended dose for patients resistant or intolerant to imatinib is 800mg/day, our patient was given 600mg/day because of residual diarrhea.

**Figure 1:**
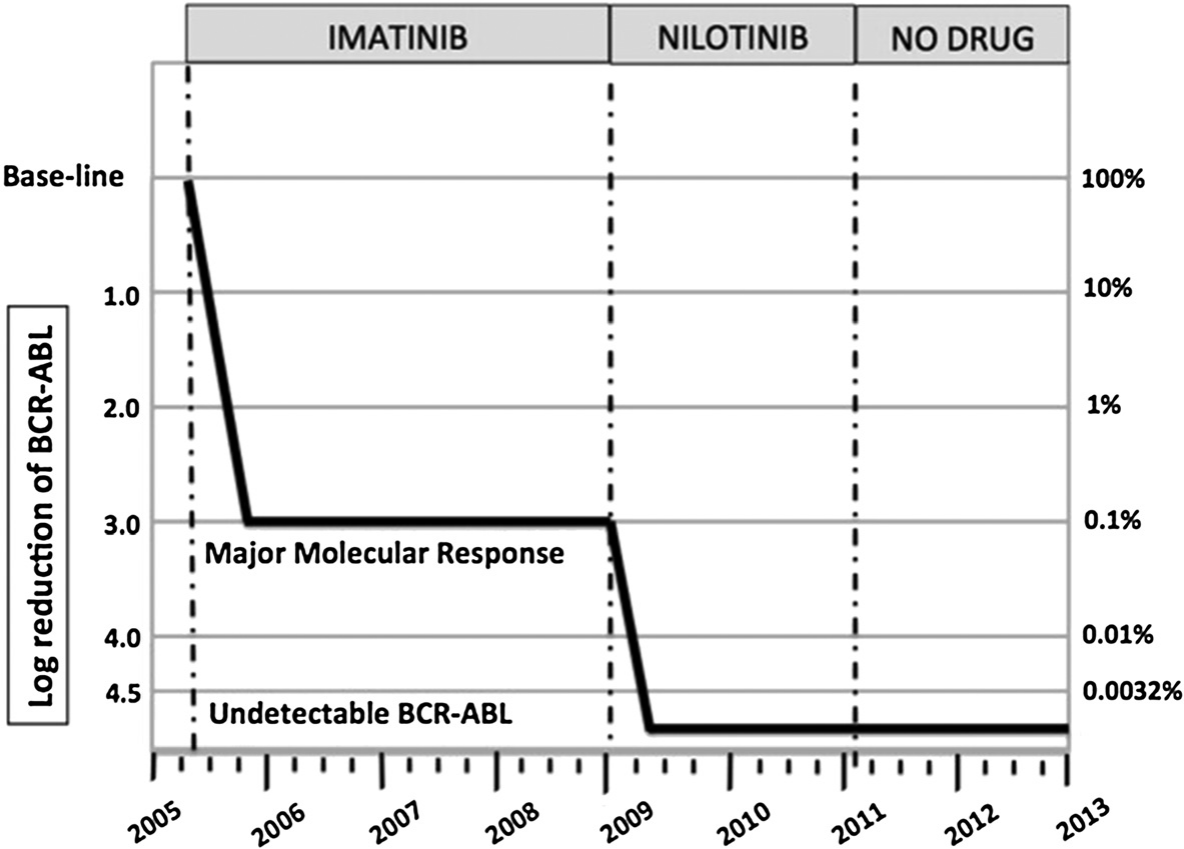
**Clinical course of the patient.** BCR-ABL levels measured during and after discontinuation of imatinib and nilotinib therapy.

He achieved early MR4.5 (BCR-ABL1≤0.0032%) and subsequently undetectable MR4.5, confirmed by nested RT-PCR (kits from Nanogen Advanced Diagnostics, Turin, Italy). The patient decided to stop nilotinib after 24 months of treatment. Undetectable MR4.5 persisted at 30 months after discontinuation of nilotinib and remains so at the time of this report (Figure 1). Investigation of the immune genetic profile of our patient revealed homozygosity for the killer immunoglobulin-like receptor (KIR) haplotype A with the following genes: *KIR2DS4*, *KIR2DL1*, *KIR2DL3*, *KIR2DL4*, *KIR3DL1*, *KIR3DL2* and *KIR3DL3*.

## Discussion

Although the ELN 2013 guidelines do not recommend discontinuation of TKI therapy in optimal responders, several attempts have been made to identify patients who can safely stop treatment [1]. The Italian consensus is that TKI discontinuation should be considered only within the context of controlled clinical trials and should be restricted to patients with MR4.5 sustained for at least 2 years. The drug needs to be promptly restarted in patients who lose MR3 [10].

In recent years, more precise definitions of international scale (IS) deep MR have been introduced: MR4 (BCR-ABL1≤0.01%), MR4.5 (BCR-ABL1≤0.0032%) and MR5 (BCR-ABL1≤0.001%). An, assay is considered negative when no BCR-ABL copies are detectable using the IS with a sensitivity ≥4.5 logs below the standardized baseline. Indeed, the term *complete molecular response* is increasingly being replaced with *undetectable molecular response*[1].

Rea *et al.*, in their study of 34 patients with CML, reported a 58% probability of maintaining a stable, deep MR at 12 months [5]. Most of their patients had previously been treated with imatinib; patients receiving second-generation TKI treatment because of intolerance to imatinib had a significantly higher probability of achieving stable undetectable MR (67%) compared to those switched to second-generation TKI because of suboptimal response or resistance (33%).

Preliminary data on 27 participants enrolled in a larger study of 63 patients with CML who stopped dasatinib after achieving undetectable MR for at least 1 year had a molecular relapse-free survival rate of 44%. Reintroduction of the drug in relapsed patients rapidly restored deep MR, and none of them progressed to advanced phase CML [6]. However, except for the study by Rea and colleagues [5] and one anecdotal case report [9], both with a median follow-up of only 1 year, there is a paucity of data on withdrawal of nilotinib in patients with CML. Interestingly, our patient was homozygous for KIR haplotype A, previously reported by our research team to be a promising immunogenetic marker for undetectable MR [11]. The authors of another recent report suggest a potential role for natural killer cells in achieving undetectable MR after discontinuation of imatinib [12].

## Conclusions

Our present report, to the best of our knowledge, is the first to describe nilotinib withdrawal with a follow-up of 30 months. Our patient was treated with imatinib for 4 years and achieved MR3 before he was started on replacement therapy with nilotinib because of intolerance. He rapidly achieved undetectable MR and, after 24 months of treatment, decided to stop the drug. He continued to have undetectable MR for 30 months after stopping the drug and remains so at the time of this report. Additional studies are recommended to confirm the feasibility and safety of discontinuing second-generation TKI therapy.

## Consent

Written informed consent was obtained from the patient for publication of this case report and any accompanying images. A copy of the written consent is available for review by the Editor-in-Chief of this journal.

## Abbreviations

BCR-ABL: Breakpoint cluster region-Abelson; CML: Chronic myeloid leukemia; ELN: European LeukemiaNet; IS: International Scale; MR: Molecular response; TKI: Tyrosine kinase inhibitor.

## Competing interests

The authors declare that they have no competing interests.

## Authors’ contributions

All authors analyzed and interpreted the patient data and wrote the manuscript. All authors read and approved the final manuscript.
